# Patient Digital Health Technologies to Support Primary Care Across Clinical Contexts: Survey of Primary Care Providers, Behavioral Health Consultants, and Nurses

**DOI:** 10.2196/32664

**Published:** 2022-02-25

**Authors:** Oleg Zaslavsky, Frances Chu, Brenna N Renn

**Affiliations:** 1 School of Nursing University of Washington Seattle, WA United States; 2 Department of Psychology University of Nevada Las Vegas, NV United States

**Keywords:** survey, primary care, acceptance, nurses, primary care providers, behavioral health consultants, mobile health, technology, health promotion, attitudes

## Abstract

**Background:**

The acceptance of digital health technologies to support patient care for various clinical conditions among primary care providers and staff has not been explored.

**Objective:**

The purpose of this study was to explore the extent of potential differences between major groups of providers and staff in primary care, including behavioral health consultants (BHCs; eg, psychologists, social workers, and counselors), primary care providers (PCPs; eg, physicians and nurse practitioners), and nurses (registered nurses and licensed practical nurses) in the acceptance of various health technologies (ie, mobile apps, wearables, live video, phone, email, instant chats, text messages, social media, and patient portals) to support patient care across a variety of clinical situations.

**Methods:**

We surveyed 151 providers (51 BHCs, 52 PCPs, and 48 nurses) embedded in primary care clinics across the United States who volunteered to respond to a web-based survey distributed in December 2020 by a large health care market research company. Respondents indicated the technologies they consider appropriate to support patients’ health care needs across the following clinical contexts: acute and chronic disease, medication management, health-promoting behaviors, sleep, substance use, and common and serious mental health conditions. We used descriptive statistics to summarize the distribution of demographic characteristics by provider type. We used contingency tables to compile summaries of the proportion of provider types endorsing each technology within and across clinical contexts. This study was exploratory in nature, with the intent to inform future research.

**Results:**

Most of the respondents were from urban and suburban settings (125/151, 82.8%), with 12.6% (n=19) practicing in rural or frontier settings and 4.6% (n=7) practicing in rural-serving clinics. Respondents were dispersed across the United States, including the Northeast (31/151, 20.5%), Midwest (n=32, 21.2%), South (n=49, 32.5%), and West (n=39, 25.8%). The highest acceptance for technologies across clinical contexts was among BHCs (32/51, 63%) and PCPs (30/52, 58%) for live video and among nurses for mobile apps (30/48, 63%). A higher percentage of nurses accepted all other technologies relative to BHCs and PCPs. Similarly, relative to other groups, PCPs indicated lower levels of acceptance. Within clinical contexts, the highest acceptance rates were reported among 80% (41/51) of BHCs and 69% (36/52) of PCPs endorsing live video for common mental health conditions and 75% (36/48) of nurses endorsing mobile apps for health-promoting behaviors. The lowest acceptance across providers was for social media in the context of medication management (9.3% [14/151] endorsement across provider type).

**Conclusions:**

The survey suggests potential differences in the way primary care clinicians and staff envision using technologies to support patient care. Future work must attend to reasons for differences in the acceptance of various technologies across providers and clinical contexts. Such an understanding will help inform appropriate implementation strategies to increase acceptability and gain greater adoption of appropriate technologies across conditions and patient populations.

## Introduction

Primary care plays a central role in managing acute and chronic physical and behavioral health conditions, including patient self-management of such conditions [1]. Digital health, which refers to the use of telehealth, mobile devices, and other wireless technologies to support health care [2], has great potential to augment or enhance such care by supporting behavior change while minimizing barriers such as distance and time. These technologies are intended to enhance education and awareness; support diagnosis and treatment, including self-management; facilitate remote monitoring; and enable remote communication (eg, telehealth) [3].

However, such technologies are far from having established maturity or wide acceptance in primary care [4]. COVID-19, the disease caused by the novel coronavirus (SARS-CoV-2), prompted drastic changes in primary care delivery, including the sudden and unexpected implementation of new tools to expand and support patient care, such as telehealth [5-7]. Innovative digital solutions such as live video, instant chats, and text messages have become essential to continue delivering primary care in times when in-person visits are restricted. Despite nationwide regulatory and reimbursement policy changes concerning health care technologies during the COVID-19 pandemic (eg, less restrictive policies for remote office visits), the implementation of digital tools may have varied across sites, providers, and clinical situations. Disparities in the adoption of digital tools might be due to differences in health care provider perception of acceptability, appropriateness, and feasibility of technologies for specific clinical scenarios [8].

Many studies have examined the acceptance and feasibility of digital health technologies for managing patients’ physical and mental health conditions. Notably, an important condition for implementing such technologies is provider attitudes, including acceptance [9]. Indeed, a recommendation from a trusted health care provider is imperative for patients to adopt technologies like mobile apps; however, health care providers’ acceptance of technologies varies [10].

Prior work examined mental health professionals’ attitudes and interests in using technology in clinical treatment, but this was restricted to websites and mobile apps [9]. Other studies gathered health care providers’ (pharmacists, physicians, and advanced practice registered nurses [APRNs]) opinions regarding the use of mobile apps for patients across various clinical contexts [11,12]. Pharmacists tended to recommend mobile apps for smoking cessation, physical activity, diabetes, weight management, and sexual health [11]. By contrast, physicians and APRNs recommended mobile apps for tracking physical activity, diet, and sleep; however, these providers did not view mobile apps as beneficial for monitoring sleep [12].

As primary care medical and behavioral health providers and staff differ in training, experience with technology, and clinical orientation, it is also reasonable to expect differences by provider type in their attitude toward digital health technologies. As such, the purpose of this project was to explore the extent of potential differences between major groups of providers and staff in primary care, including behavioral health consultants (BHCs; eg, psychologists, social workers, and counselors), primary care providers (PCPs; eg, physicians and nurse practitioners), and nurses (eg, registered nurses and licensed practical nurses) in their acceptance of different technologies to support patient care across a variety of clinical situations. This study was exploratory in nature, with the intent to inform future research.

## Methods

### Study Design and Sampling

We surveyed 151 providers (51 BHCs, 52 PCPs, and 48 nurses) embedded in primary care clinics across the United States who volunteered to respond to a web-based survey invitation. Survey methods are reported in accordance with the Checklist for Reporting Results of Internet E-Surveys (CHERRIES) [13]. Recruitment was overseen by a large health care market research company. The company emailed the survey invitation to their proprietary panel of health care professionals in December 2020. Invitations were unique to each participant to avoid duplicate responses and to coordinate incentive payment through the market research company. The invitation link directed interested participants to our web-based survey portal for screening and participation. Responses were collected and managed using the Research Electronic Data Capture (REDCap) web tool [14]. Screening questions asked participants to verify that they worked in primary care and to select their role (BHC, PCP, nurse, or other; the latter were excluded). Given the preliminary nature of our data collection, we applied a quota of approximately 50 respondents per provider group. Respondents were required to select an answer for all items to complete the survey; however, we included options such as “prefer to not answer” (for potentially sensitive items such as demographics) and occasionally included “unsure/don’t know” for select relevant items.

### Ethics Approval

The University of Washington Human Subjects Division determined that the survey was not considered research, as defined by federal and state regulations; therefore, no review by the institutional review board was required and participants did not have to provide informed consent. Eligible participants were presented with a basic description of the study and asked to indicate agreement to participate before advancing. No personal or identifying information was collected. Participants received a monetary incentive for their time.

### Measures

The parent survey aimed to examine provider use of technologies to support behavioral health since the onset of the COVID-19 pandemic. The main outcome in the present study was provider acceptance of digital health technologies in primary care, based on responses to a single question. This item asked the respondents to select technologies they consider appropriate to support patients’ health care needs in the following clinical contexts: acute and chronic disease, medication management, health-promoting behaviors (diet and physical activity), sleep, substance use (eg, alcohol, nicotine), common mental health disorders (eg, depression, anxiety), and serious mental health conditions (eg, schizophrenia, bipolar disorder). The exact wording of the question was “What technologies can you envision to support behavioral and lifestyle changes in your patients?” Possible technologies included mobile apps, wearables, live video (clinical visits via interactive video), phone, email, instant chat, text messages, social media, and a patient portal. Respondents were presented with a matrix of possible technologies across the 8 clinical contexts and could mark as many technologies as they deemed appropriate within each context.

### Statistical Analysis

We used descriptive statistics to summarize the distribution of demographic characteristics by provider type. Contingency tables compiled summaries in terms of a proportion of providers by type endorsing technologies within and across all contexts.

## Results

The respondents included 51 BHCs, 48 nurses, and 52 PCPs. Most were located in urban and suburban settings and were dispersed across regions of the United States. See Table 1 and Multimedia Appendix 1 for the participants’ demographic details.

**Figure 1:**
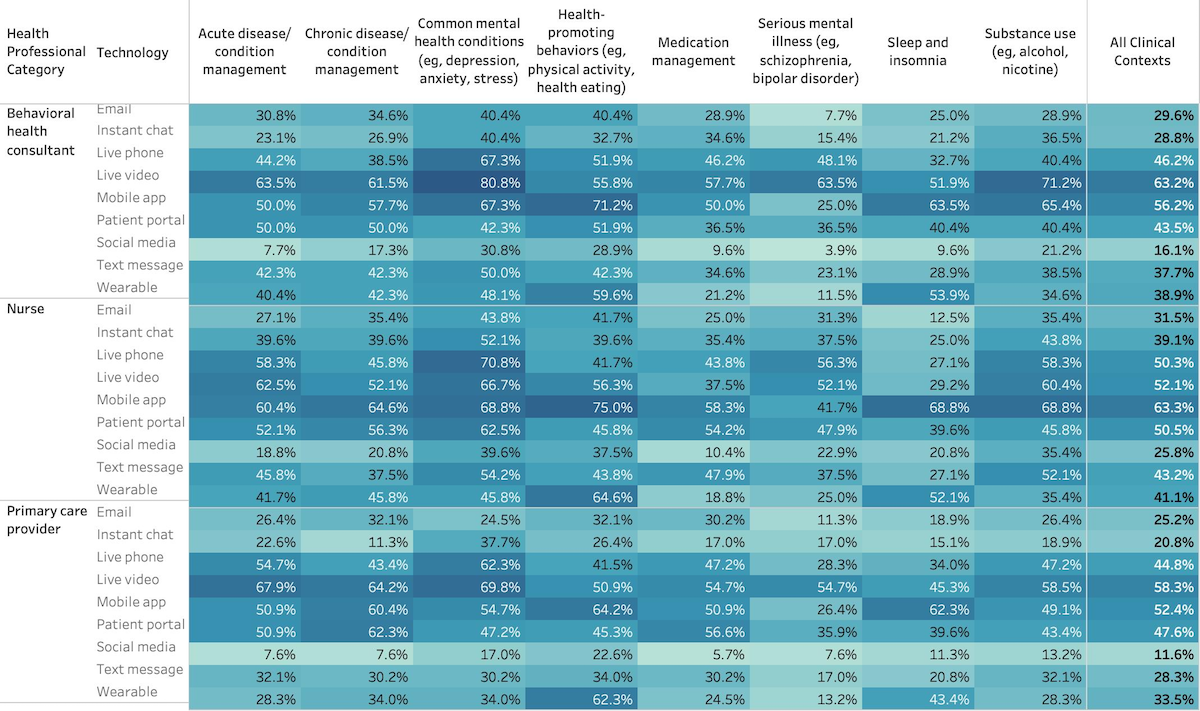
Acceptance of digital health technologies across various clinical contexts by major primary care health care professional types (n=51 behavioral health consultants, n=52 primary care providers, and n=48 nurses).

**Figure 2:**
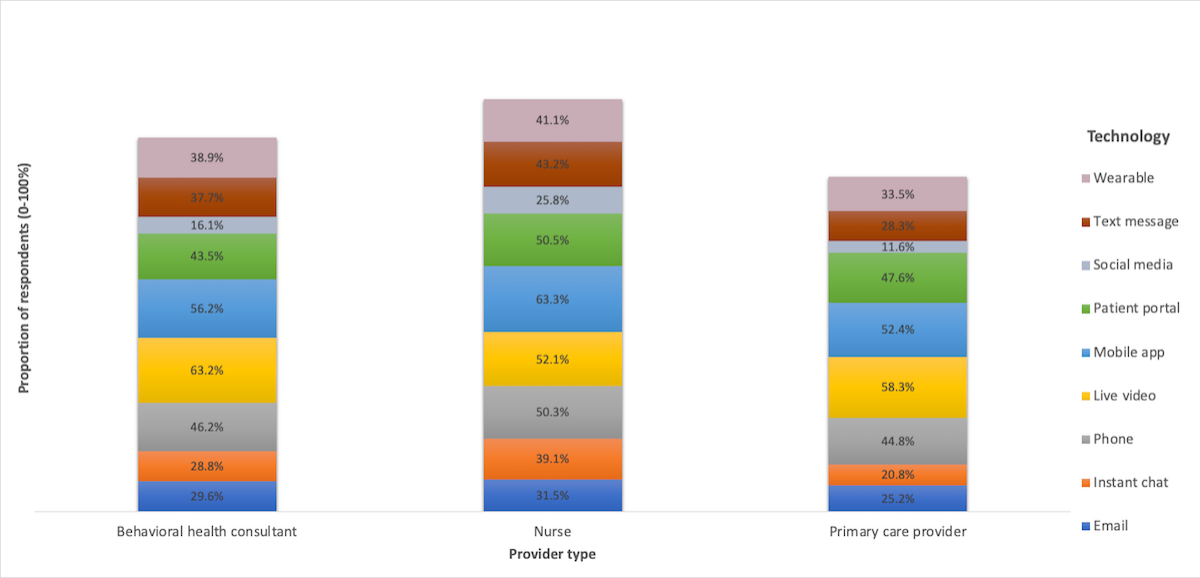
Proportion of respondents endorsing a specific digital health technology across all clinical contexts (n=51 behavioral health consultants, n=52 primary care providers, and n=48 nurses).

We observed potential differences by provider type in the acceptance of technologies within and across all clinical contexts. The highest acceptance rates for technologies across clinical contexts were among BHCs for live video (32/51, 63%), nurses for mobile apps (30/48, 63%), and PCPs for live video (30/52, 58%). In addition to their support for mobile apps, a greater proportion of nurses accepted all other technologies relative to BHCs and PCPs. More than half of the nurse respondents endorsed synchronous technologies such as phone calls or live video, as well as the patient portal. Relative to other groups, PCPs had lower rates of acceptance. Within clinical contexts, the highest acceptance rates were 80% (41/51) of BHCs and 69% (36/52) of PCPs endorsing the use of live video for common mental health conditions and 75% (36/48) of nurses endorsing mobile apps for health-promoting behaviors. Endorsement of technologies was variable, but generally low for serious mental illness across provider types. The lowest acceptance across providers was for social media in the context of medication management (9.3% [14/151] endorsement across provider type). See Figure 1 and Figure 2 for more detail. Figure 1 illustrates the proportion of respondents endorsing a specific technology. The least (lightest blue) to most opaque (darkest blue) shades represent low to high values (0% to 100%). Figure 2 illustrates the proportion of respondents endorsing a specific technology across all clinical contexts.

**Table 1:** Demographic characteristics of behavioral health consultants, nurses, and primary care providers who participated in the study.

Characteristic		Behavioral health consultants (n=51)	Nurses (n=48)	Primary care providers (n=52)	Total sample (N=151)
**Race, n (%)**					
	Black or African American	3 (6)	3 (6)	2 (4)	8 (5.3)
	American Indian or Alaska Native	0 (0)	0 (0)	1 (2)	1 (0.7)
	Asian	1 (2)	5 (10)	13 (25)	19 (12.6)
	Native Hawaiian or Other Pacific Islander	0 (0)	0 (0)	0 (0)	0 (0)
	White	45 (88)	33 (69)	30 (58)	108 (71.5)
	More than one race	1 (2)	1 (2)	0 (0)	2 (1.3)
	Prefer to not answer	1 (2)	6 (13)	6 (12)	13 (8.6)
**Ethnicity, n (%)**					
	Hispanic/Latinx	3 (6)	8 (17)	3 (6)	14 (9.3)
	Not Hispanic/Latinx	46 (90)	35 (73)	45 (87)	126 (83.4)
	Prefer to not answer	2 (4)	5 (10)	4 (8)	11 (7.3)
**Age in years**					
	Mean (SD)	51.7 (11.9)	47.1 (11.4)	45.1 (10.8)	48.0 (11.7)
	Range	30-73	24-67	29-77	24-77
**Gender, n (%)**					
	Female	37 (73)	30 (63)	24 (46)	91 (60.3)
	Male	13 (26)	9 (19)	24 (46)	46 (30.5)
	No response	1 (2)	9 (19)	4 (8)	14 (9.3)
**Clinic setting, n (%)**					
	Urban	21 (41)	17 (35)	13 (25)	51 (33.8)
	Suburban	22 (43)	22 (46)	30 (58)	74 (49)
	Rural	4 (8)	4 (8)	9 (17)	17 (11.3)
	Rural-serving	3 (6)	4 (8)	0 (0)	7 (4.6)
	Frontier	1 (2)	1 (2)	0 (0)	2 (1.3)
**Clinic type, n (%)**					
	Clinic/practice at an academic medical center	5 (10)	10 (21)	10 (19)	25 (16.6)
	Clinic/practice affiliated with a university teaching hospital	3 (6)	6 (13)	7 (14)	16 (10.6)
	Community health center and/or Federally Qualified Health Center	6 (12)	5 (10)	7 (14)	18 (11.9)
	Private health care system	8 (16)	14 (29)	14 (27)	36 (23.8)
	Veteran’s Affairs medical center or community-based outpatient clinic	2 (4)	2 (4)	0 (0)	4 (2.6)
	Other government hospital	2 (4)	1 (2)	0 (0)	3 (1.98)
	Private (independent or group) practice	29 (57)	11 (23)	17 (33)	57 (37.7)
	Other	1 (2)	3 (6)	1 (2)	5 (3.3)

## Discussion

This national survey suggests potential differences in the way primary care clinicians, behavioral health consultants, and nursing staff envision using digital health technologies to support patients in primary care. Potential differences were observed across technologies and clinical contexts. Compared to other providers, a higher proportion of BHCs in our sample were receptive to using synchronous interactive technologies such as live video. Some of this acceptability may be a result of the sudden shift to telehealth during the COVID-19 pandemic [15]. Similarly, a high proportion of nurse respondents embraced more diverse ways of connecting and supporting patients through traditional technologies such as phone and email, as well as newer technologies such as mobile apps and instant chats. Relative to BHCs and nurses, PCPs in our sample indicated lower levels of acceptance for digital health technologies across all clinical situations. Across clinical contexts, live video was seen as an acceptable way of connecting with patients, especially for common mental health conditions. Mobile apps earned high acceptance among nurses, especially in the context of health-promoting behaviors. In general, the acceptance of social media lagged behind other technologies. It is possible that the nature of patient interaction in primary care influenced provider attitudes. Nurses endorsed technologies that support their typical clinical focus on case management, self-management, and promoting health behaviors. By contrast, PCPs and BHCs preferred synchronous video, which aligns with their focus on traditional treatment encounters. We recommend researchers and developers solicit provider needs and preferences when designing digital health technologies to promote the usability and implementation of these tools.

Given that we collected data during the first year of the COVID-19 pandemic, our findings may reflect this pivotal moment for the adoption of digital health tools to provide or enhance care. The nationwide rollout of digital technologies (eg, electronic health records) to support patient care has often faced challenges, and policymakers often struggle to understand how, when, and to what extent technologies could be used. Our preliminary findings highlight potential differences in the acceptance of digital health technologies across providers and clinical situations. Given that acceptance and other attitudinal constructs are considered preconditions for adoption [8], a one-size-fits-all approach to introducing technologies may fail among different providers. Understanding the reasons for such observed differences in acceptance—that is, exploring why the differences exist, perhaps through a qualitative investigation—is an important future direction.

Our study has several limitations. First, the convenience sample may not be representative of all US providers and staff in primary care. Nonresponders may have different opinions about digital health technologies across clinical contexts, while responders may be biased toward using technology in any clinical context. Secondly, the survey was not a validated measure of technology acceptance. Thirdly, our findings are based on a cross-sectional survey that reflects one point in time in the midst of a global pandemic. Longitudinal follow-up is necessary to better ascertain trends in technology acceptance. Fourth, we did not provide context for how technologies would be employed and by whom. Finally, because our sample size is small relative to the number of response items (technologies and clinical contexts), we present descriptive findings and comparisons rather than statistical testing of group differences.

In conclusion, given the potential of technologies to facilitate primary health care delivery, future work must attend to reasons for differences in acceptance of various technologies across providers and clinical contexts. Such an understanding will help inform appropriate implementation strategies to increase acceptability and gain higher adoption rates of appropriate technologies across conditions and patient populations.

## Appendix

Multimedia Appendix 1 Demographic characteristics of health care professionals who participated in the survey, including additional data points.

## Abbreviations

APRN: advanced practice registered nurse
BHC: behavioral health consultant
CHERRIES: Checklist for Reporting Results of Internet E-Surveys
PCP: Primary care providers
REDCap: Research Electronic Data Capture
