# Supplementary material for: Patient Digital Health Technologies to Support Primary Care Across Clinical Contexts: Survey of Primary Care Providers, Behavioral Health Consultants, and Nurses
Source: JMIR Form Res. 2022 Feb 25;6(2):e32664. doi: 10.2196/32664 (PMC8917439; doi:10.2196/32664)
Supplement: Multimedia Appendix 1 [file formative_v6i2e32664_app1.docx]

**Supplemental Table 1:** Healthcare Professional Participant Characteristics (Additional Datapoints)

|  | Behavioral Health Consultants (BHCs)  *n* = 51 | Nurses  *n* = 48 | Primary Care Providers (PCPs)  *n* = 52 | Total Sample  (*N* = 151) |
| --- | --- | --- | --- | --- |
| Profession/Training, *n* (%) |  |  |  |  |
| Licensed clinical social worker (LCSW) | 17 (33.3%) | -- | -- | 17 (11.3%) |
| Psychologist (PhD/PsyD) | 14 (27.5%) | -- | -- | 14 (9.3%) |
| Licensed counselor (LMHC/LPC) | 9 (17.6%) | -- | -- | 9 (5.96%) |
| Licensed marriage and family therapist (LMFT) | 5 (9.8%) | -- | -- | 5 (3.3%) |
| Other social worker | 4 (7.8%) | -- | -- | 4 (2.6%) |
| Substance use professional | 3 (5.9%) | -- | -- | 3 (1.98%) |
| Psychiatric nurse practitioner | 1 (1.96%) | -- | -- | 1 (0.7%) |
| Other behavioral health | 1 (1.96%) | -- | -- | 1 (0.7%) |
| Registered nurse (RN) | -- | 40 (83.3%) | -- | 40 (26.5%) |
| Licensed practical nurse (LPN) | -- | 8 (16.7%) | -- | 8 (5.3%) |
| Physician (MD/DO) |  |  | 47 (90.4%) | 47 (31.1%) |
| Nurse practitioner | -- | -- | 5 (9.6%) | 5 (3.3%) |
| Trainee Status, *n* (%) |  |  |  |  |
| No | 46 (90.2%) | 46 (95.8%) | 50 (96.2%) | 142 (94.0%) |
| Yes | 5 (9.8%) | 2 (4.2%) | 2 (3.9%) | 9 (5.96%) |
| Patient Panel Size |  |  |  |  |
| Mean (SD) | 422.6 (1435.9) | 1909.4 (4153.1) | 2525.1 (2077.3) | 1559.4 (2856.5) |
| Range | 6-10000 | 10-20000 | 6-9000 | 6-20000 |
| Geographic Location, *n* (%) |  |  |  |  |
| Northeast (PA, NY, NJ, CT, RI, MA, VT, NH, ME) | 13 (25.5%) | 12 (25.0%) | 6 (11.5%) | 31 (20.5%) |
| Midwest (ND, SD, NE, KS, MN, IA, MO, WI, IL, IN, MI, OH) | 8 (15.7%) | 12 (25.0%) | 12 (23.1%) | 32 (21.2%) |
| South (TX, OK, AR, LA, MS, AL, GA, FL, SC, NC, TN, KY, WV, VA, MD, DC, DE) | 12 (23.53%) | 13 (27.08%) | 24 (46.15%) | 49 (32.5%) |
| West (WA, OR, CA, ID, NV, MT, WY, UT, CO, AZ, NM, AK, HI) | 18 (35.29%) | 11 (22.92%) | 10 (19.23%) | 39 (25.8%) |
| Medicare/Medicaid Percentage |  |  |  |  |
| Mean (SD) | 41.9 (34.8) | 56.5 (26.4) | 47.1 (23.2) | 48.1 (29.2) |
| Range | 0-100 | 0-100 | 5-95 | 0-100 |
| Behavioral/Mental Health Integration in Primary Care Clinic, *n* (%) |  |  |  |  |
| Fully integrated behavioral health | 20 (39.22%) | 14 (29.17%) | 7 (13.46%) | 41 (27.2%) |
| Partial integrated behavioral health | 16 (31.37%) | 20 (41.67%) | 15 (28.85%) | 51 (33.8%) |
| Available behavioral health | 2 (3.92%) | 6 (12.50%) | 10 (19.23%) | 18 (11.9%) |
| No integrated behavioral health | 9 (17.65%) | 8 (16.67%) | 20 (38.46%) | 37 (24.5%) |
| Other | 4 (7.84%) | -- | -- | 4 (2.6%) |
